# Understanding the Chinese Hui Ethnic Minority’s Information Seeking on Cardiovascular Diseases: A Focus Group Study

**DOI:** 10.3390/ijerph16152784

**Published:** 2019-08-04

**Authors:** Lei Yang, Yuping Mao, Jeroen Jansz

**Affiliations:** 1 Erasmus Research Centre for Media, Communication and Culture, Erasmus School of History, Culture and Communication, Erasmus University Rotterdam, 3062 PA Rotterdam, The Netherlands; 2 Department of Communication Studies, College of Liberal Arts, California State University Long Beach, Long Beach, CA 90840, USA

**Keywords:** the Chinese Hui ethnic minority, culture, cardiovascular diseases, health information, focus group, needs

## Abstract

The Chinese Hui ethnic minority group is an Islamic minority. The Hui people comprise the third largest minority population in China and are widely distributed throughout the country. Previous research shows that the Hui had a higher prevalence of cardiovascular risk factors (CVRFs) than most other ethnic groups. Therefore, the availability of health information relating to these factors is especially important for the Hui minority’s preventive healthcare. They do, however, experience difficulties in obtaining health-related information. The current research aims to identify the needs of the Hui people on where and how they obtain cardiovascular disease (CVD) related information from the media and other sources. Six focus groups were conducted in Shenyang City. The results revealed that the participants relied on different sources to get advice about CVDs, of which the internet and television were the most prominent ones. The participants expressed a desire for credible and professional information from different sources and asked for mediated health communication programs specifically targeted at the Hui. In addition, the participants felt ignored by the Chinese mainstream media at large, which created barriers for them to get health information.

## 1. Introduction

The Chinese Hui people are an Islamic minority in China. Their health situation is unique because they experience a higher prevalence of cardiovascular risk factors (CVRFs) than the Chinese Han majority and most other minority groups [1]; in particular, they suffer from a high prevalence of hypertension [1]. Thus, the Hui people need preventive health information to reduce their chance of getting cardiovascular diseases (CVDs). However, they experience difficulties in obtaining health-related information. There are indications that inequalities exist across different ethnic groups in obtaining health information [2,3]. In some cases, the health information targeting at minorities was simply lacking; in other cases, the minorities could not access available information [2,4]. Previous research has shown that, in multicultural societies, disadvantaged groups are more likely to use media (e.g., the internet) to acquire information (e.g., health information) to overcome existing social inequalities in accessing information [5].

In China, there exist fifty-six ethnic groups that are identified by the central government: 55 ethnic minority groups and the Han majority [6,7]. Although the 55 minority groups host a population of 114 million people, which accounts for 8 percent of the whole population in China [8], there is limited research about how Chinese minorities obtain health information in the multicultural society. In particular, there is hardly any health communication research related to CVDs conducted among the Chinese Hui people, despite the size of Hui population (around 10 million) [9]. Thus, this paper aims to contribute to filling that lacuna by investigating what health information the Hui need about CVDs and what they have obtained. First, the theoretical framework for the focus group study is discussed. Next, we describe how we organized and executed the focus groups and how we analyzed what was shared therein. The presentation of the results is organized according to the four overarching themes that emerged from our analysis. Finally, we reflect and discuss the results in the light of previous academic literature and the specific social context of the Hui in China. Based on the strengths and limitations of this study, we propose future research directions in this line of research. Besides, this Chinese minority case can also help us understand that minorities have specific needs related to their position in a multicultural society, and these needs can partly be fulfilled by media.

### 1.1. The Chinese Hui People’s Socio-Cultural Background

This paper focuses on the Chinese Hui ethnic minority, which is the third largest minority group in China and is also widely distributed throughout the country [10]. At first glance, the Chinese Hui are quite similar to the Han, the Chinese ethnic majority, because the Hui share customs, language, and culture with the Han [11]. On closer inspection, the Chinese Hui have their own ethnic culture, because many of them follow Islamic dietary laws and take part in religious activities [12]. Previous research shows that health information that is culturally appropriate for a specific group is more effective than information that does not integrate culture [13]. It is, therefore, relevant to know what the Hui need in terms of health information in a multicultural environment.

In order to understand the Chinese Hui minority’s health, it is important to consider the association between dietary habits and health. It is evident that the traditional dietary habits of a cultural group can promote or prevent certain diseases [14]. For example, dietary habits are among the strongest predictors of CVRFs [15]. As believers in Islam, the Hui have different dietary habits compared to the majority of the Chinese population. In particular, the Hui people like to eat high-sugar and high-fat food such as beef, mutton (including inners), and to drink Babao tea [16]. These foods can cause high prevalence of hyperlipidemia among the Hui [16]. In addition, previous research shows that high sugar dietary habits can increase the incidence of CVDs [17]. Thus, the Hui’s unique dietary habits can make them susceptive to CVDs.

CVDs are currently the leading cause of death in China [1,18,19]. Hypertension, diabetes, dyslipidemia, overweight/obesity, and current smoking are known to be the five main CVRFs [1,18,19,20] that can cause CVDs. With the higher prevalence of CVRFs among the Hui minority than the Chinese Han majority and most other minority groups, it is important to come up with effective intervention plans for the Hui. Health education containing information about a healthy diet and lifestyle can be effective in reducing CVRFs. It is, therefore, crucial to gain insight into how the Hui people obtain health information related to CVDs.

In this research, we focus on the circumstances of the Hui in an urban area, namely Shenyang City, because Shenyang is the biggest city in Northeast China, and there is one area called “回回营 (In English: Huihui Quarter)” where most Hui people live in Shenyang City.

### 1.2. Cultural Influences on Health Communication

China is a country with many ethnicities and diverse cultures [21]. In multicultural societies, cultural factors play an important role in health communication because a group’s cultural characteristics can be related to health decisions [22,23], behavior, or the adoption of health-education and health-communication programs [14,24,25]. Thus, culture needs to be taken into account in health communication [26].

Health communication in cultural backgrounds can be understood in two approaches: the culture-centered approach and the cultural sensitivity approach [26]. The culture-centered approach (CCA) emphasizes social structures surrounding health can be changed through dialogues with cultural members, which can create spaces for minorities’ cultural voices [26]. In China, the Han culture is dominant. Ethnic minority groups’ cultures are different from the Han culture, but the information from media mainly targets the Han majority. Based on the framework of CCA, the dialogues with ethnic minority groups can be treated as alternative ways to listen to their voices and bring their narratives into mainstream structures, which enable the access to health resources [27]. Thus, we conducted this focus group study to get to know the Hui’s needs for CVD health information.

Taking cultural differences into consideration, the cultural sensitivity approach presents that there should be appropriate health messages tailored to the cultural factors of specific groups [26]. First, it is necessary to identify the groups experiencing poor health from a specific medical perspective (e.g., the Chinese Hui people have a high prevalence of CVRFs), and then to seek to recognize the cultural factors that influence health in these particular groups (e.g., dietary habits). Utilizing this approach, a specific group’s cultural characteristics, values, and behaviors can be recognized, enhanced, then relied on to provide targeted health information. This approach has been suggested as a practical way to address culture in health communication [14,24]. Health communication, as such, can employ different sources, which is discussed in the next section.

### 1.3. Health Information from Different Sources

Health information is essential because knowledge about health and disease may help to keep individuals well by encouraging them to adopt a healthy lifestyle. This was demonstrated by Street and Piziak [28], who found that the successful treatment of chronic disease relied mainly on the patients’ ability to lead a healthy lifestyle, taking medications as required, and reducing risk factors (e.g., by losing weight). Health information can also be used to understand the diagnosis and treatment options for various diseases [29,30,31], as well as disease prevention [4].

Individuals can obtain health information from various sources. Key informants are medical professionals [29,32], other healthcare providers [29], and interpersonal networks of family, friends, and colleagues [33,34,35]. In addition to health information from these sources, individuals often find valuable assistance in both traditional media (newspapers, magazines, radio, and television) [33,36] and online media, including social media [37,38,39,40]. Media serve an important role in bridging information gaps in multicultural societies [41]. Mediated information is particularly relevant because individuals can follow their own preferences for mediated sources: they can watch a health program on television or actively search online and check many different health-related websites. Online health information has become one of the most important information sources globally in recent years [30,42,43]. Furthermore, obtaining health information via social media (e.g., WeChat) is now very common in China [40]. The Hui people can simultaneously check many different sources, thus gaining knowledge about multiple examples. They can then choose which information is most suitable for their particular circumstances. Accordingly, we assume that the Hui people may obtain different kinds of health information from different sources. Our first research question targets the relevance of health information to the Hui by asking:

RQ1. What kind of health information on CVDs do the Hui people obtain from different sources?

### 1.4. Health Information Needs

Our second research question is concerned with the reasons why Hui people seek health information related to CVDs. Seeking specific information is goal-directed behavior that stems from needs, which can be different and related to different aspects of being unwell. Previous research found that health information must satisfy two different needs: 1) cognitive needs—concerned with (factual) information about disease prevention and treatment, and 2) emotional needs—namely needs that concern feelings related to a disease and its consequences [30]. Research by Tustin (2010) [44] indicates that individuals who seek information about particular medical conditions have specific needs related to: diagnosis [45,46]; prognosis [30,46]; treatment options [30,45,46,47,48,49,50]; the side-effects of treatment [30,47]; and getting appropriate support [30,45,46,48,49]. Health information needs are prevalent and diverse; for example, previous research has shown that individuals with diabetes or a CVD indicated that they hoped to be better informed about the risks and effects of their diseases when they were diagnosed [51]. Fulfilling that need would have helped them to pay more attention to the consequences of their unhealthy lifestyles at that time [51]. Accordingly, the current study also aims to answer the following:

RQ2. What needs do the Hui people have relating to health information about CVDs from different sources?

## 2. Materials and Methods

This study employed a focus group methodology to identify the needs of the Hui people with respect to CVD health information from different sources, with a particular focus on how the participants appraised the quality of such information and how it satisfied their needs. Focus groups have been widely applied to explore minority groups’ health experiences. For instance, focus groups have been conducted to understand immigrant minority people’s attitudes, knowledge, and health-seeking behaviors in the prevention and screening of cancer [52]. Lunt and Livingstone (1996) emphasized that a focus group can be seen as a context in which everyday communication, which is inaccessible for researchers, is simulated [53]. A major strength of focus groups is the interaction between members [54]. Participants respond to each other in the conversation, which may create an awareness of issues that they may not have considered before and even solutions to a problem [54]. The outcomes may also inductively result in findings that were not anticipated by researchers. Focus groups provide new and rich information in early groups; later groups may provide overlapping information from early groups [53]. The re-occurring themes across different groups reveal shared experiences and perspectives. In this research, six focus groups, each with three to four participants, were conducted in Shenyang City. In total, there were 23 Hui participants, including nine men and 14 women aged between 23 and 75. The focus groups lasted for, on average, 60 min. A snowball technique was used to recruit the participants, with local support from a mosque and a Hui primary school. The focus groups were conducted in Mandarin, and the transcripts were translated into English.

Previous research has shown that a focus group runs best when the participants know each other, and the groups take place in an everyday setting that is familiar [55,56]. Accordingly, the groups were composed in such a way that each member knew one or two other participants. The focus groups took place in familiar settings, like the workplace or a participant’s home. One of the authors moderated the discussion. To enable the content of the focus groups to be analyzed thoroughly, the moderator recorded the proceedings using two recording devices. The moderator informed all the participants in advance about this procedure and asked them to sign letters of consent and complete a short questionnaire about their demographic characteristics (see Table 1), dietary habits, and health status. At the end of the session, the participants were given a small gift as a token of appreciation.

The answers to the questionnaires showed that 12 participants only ate Halal food and never drank alcohol. Six others followed most Hui eating habits by generally preferring Halal food and not drinking alcohol. One participant only partially conformed to the relevant dietary habits by not eating pork, but still drank alcohol. Four others did not follow the Hui eating habits at all and ate pork. Referring to their health status, only 13 out of 23 participants described themselves as healthy without any CVDs or CVRFs.

All the focus groups unfolded according to a similar procedure. First, the moderator welcomed the participants to the focus group. Next, she explained the purpose of the focus group as enabling her to acquire data for her PhD research on health communication issues among the Chinese Hui ethnic minority people in the city of Shenyang, which will contribute to improving knowledge about health communication targeted at the Hui. She then gave an overview of the topic and provided guidelines for the discussion. Thereafter, she initiated the discussion by asking the participants to write down all the sources they used to obtain information about CVDs. Five minutes later, the participants were asked to place their papers in a position where everyone could see and discuss them during the focus group. In order to start the conversation about the health information they had obtained, the moderator then asked the group to share what kind of CVD health information they had gleaned from the sources they had written down. In the final part of the discussion, the moderator also asked the participants to write down what kind of CVD-related health information they needed most.

A thematic analysis was used to analyze the transcripts because this is a systematic procedure with enough flexibility to allow for (theoretical) freedom in the interpretation [57]. The authors analyzed the transcripts using inductive analysis techniques, which require all the themes of the analysis to emerge from the data [52]. The coding proceeded stepwise. The first step was ‘open coding’, in which the coder read through all the transcripts and coded the themes inductively. After open coding, axial coding was applied to draw themes from the transcripts. Finally, we conducted a selective coding procedure and chose representative quotes from the participants for different themes.

## 3. Results

Four themes were identified in the thematic analysis of the data: (1) CVD health information obtained from different sources; (2) the credibility of health information about CVDs; (3) the Hui’s neglected feelings; and (4) the Hui’s needs relating to CVDs. These themes will now be explained in detail.

### 3.1. Cardiovascular Disease Health Information Obtained from Different Sources

The information sources that the participants wrote down at the start of each focus group revealed that the Hui people used different sources to acquire CVD-related health information. Table 2 shows that the internet and television were the most prominent channels. During the discussions, three participants also mentioned WeChat, a very popular social media platform in China, as a source. Next, the participants were asked about the kind of CVD health information they were looking for from different sources. In response, they shared that they mainly wanted to obtain information about prevention and treatment for both themselves and their family members. None of the participants had ever heard of CVD health information from any source that targeted the Hui.

The discussion then moved on to why the participants were so focused on obtaining CVD health information regarding prevention and treatment. In summary, they thought it was necessary to obtain some basic medical knowledge of CVDs because of their high prevalence in China. Lisa, a 47-year-old female teacher, set out her opinions:

“I mainly got information about treatment and prevention for myself and also for my family. I think it’s necessary for people to know how to prevent CVDs, and this comes from common medical knowledge. Besides, we need to know which medicines we can use when there’s an emergency, and what medicines are reasonable.”

Four of the participants mentioned that they would probably put more time and effort into obtaining information about CVDs if family members were affected. Not only did they pass the relevant information on to family members, but they also used it to stop themselves from developing CVDs, because they were concerned that they would be affected due to their genes. Ian, a 23-year-old male bachelor student, presented his fear as follows:

“I get CVD health information mainly from the internet, but I’ve got some targets. Because our family has hereditary hypertension, I pay attention to the prevention aspect. The hereditary of hypertension is obvious, so I need to know the symptoms and then how to prevent hypertension.”

When asked about whether they adjusted their behaviors according to the information they obtained, the participants all said they were not able to follow all of the advice. One reason for this was their working/living conditions (e.g., no place to keep fit by exercising), while other objective circumstances (e.g., cold weather in winter) were also a factor. Another reason was that some participants considered themselves to be so busy with their work and family that they did not have time to do as much physical activity as the information recommended. In addition, participants mentioned that when they did have some free time, they preferred to use it to engage in relaxing activities rather than focusing on their own health. Mia, a 30-year-old female office worker, said:

“I can’t follow all the health information related to preventing CVDs. I can do no smoking, no drinking, and less salt. But in terms of doing sports more, one reason I can’t is that it’s super cold in winter in Shenyang… two subjective reasons: firstly, I don’t have enough time; secondly, I’m kind of lazy. So I can only partly do as some of the information suggests.”

Next, the participants were asked whether they were satisfied with the CVD health information they had obtained from different sources. Among these 23 participants, seven said they were satisfied, and 15 were unsatisfied. Nancy, a 48-year-old female accountant, said that she was satisfied with the quantity of health information she had acquired, but not with its quality. Only one participant said that he did not pay any attention to advice about CVDs.

### 3.2. The Credibility of Health Information about CVDs

One of the main reasons why most of the Hui participants were not satisfied with the CVD-related information they had acquired was that they had faced obstacles when obtaining it, particularly with respect to its credibility. Most of the participants complained about the difficulty they had in identifying how credible health information was from different sources. Jasmine, a 49-year-old female teacher, expressed her view as follows:

“Now if I want to get some information, I can manage it using the internet. But the credibility is one issue, and sometimes we doubt it. We don’t know whether the information is real or fake, so we can’t trust all of it.”

The participants also had different views about the credibility of information from different sources. Mary, a 35-year-old female office worker, said:

“In China, physicians can’t provide specific explanations and may not tell the truth because of their own benefits. If I consult more, then the physicians aren’t happy. But when there’s a situation happening, we have no other choices but to follow the physicians’ suggestions.”

Other participants had similar views; they expressed their helplessness, as well the fact that they could not totally trust physicians, although they still thought that doctors were more professional and knowledgeable and so they tried to have confidence in them and follow their suggestions. Some of the participants were puzzled by the contradictory information expressed by the same media. Nancy, a 48-year-old female accountant, shared two typical stories:

“On the Liaoning TV channel, a health program called Health Body Light once mentioned that eating sweet potatoes was good and can cure some diseases. After a while, the program changed by saying that eating sweet potatoes was not good. So, the program had two contradictory recommendations, and we don’t know which one to follow.”

Later in the discussion, she gave another example:

“My husband bought me two tons of protein powder; then I checked online about how to use it, and I found that there were like 100 pieces of information with 100 pieces of advice. Some of the information said that you could eat it, but some said that this was not suitable to eat; so, I don’t know which one to believe.”

We found that what troubled the participants most was that they were eager to obtain appropriate health information, but could not determine which advice was the most trustworthy. The participants’ distrust of Chinese medical information was so strong that a few said that they preferred to rely on information from other countries and they wanted to know how they could obtain information about CVDs from abroad. Mia, a 30-year-old female office worker, said:

“I want to know how to get health information about CVDs from other countries. What we can get online is health information from China, but we can’t get information from other countries.”

Later, Mia also addressed why she wanted to get information from other countries:

“I trust health information from other countries more than that from China… If I get information from abroad, it can be more specific, and I can get information targeting my own situation more, but doctors in China can only explain the general situation, which has only limited relevance to my own circumstances.”

Finally, it became clear in the focus group discussions that the participants were critical of the structure of the Chinese medical system. Mia said that physicians perhaps cared more about earning money and making a profit than about their patients’ health. This was echoed by other participants, who complained that doctors cared too much about their benefits and too little about improving the health of patients. Wendy, a 53-year-old female manager, explained her views:

“There’s one serious issue in China that’s called ‘over-treatment’. If I go to hospital, even if I don’t have any serious symptoms, the doctors will still suggest a list of medicines to buy; then I have to find some friends or relatives I trust to consult with and decide whether to buy the medicines or not. This is because, sometimes, the medicines that the doctor recommends are related to his profits. It’s hard to tell whether the medicines are suitable for my condition or not.”

### 3.3. The Hui’s Neglected Feelings

The focus group participants linked their serious concerns about the credibility of health-related information and the structure of the Chinese medical system to their feelings of being ignored which impaired their satisfaction with the health information available about CVDs. It was often mentioned that the Hui people did not receive any beneficial treatment, which made them feel that they were being ignored. The participants complained, for example, that beef and mutton were much more expensive than pork, but the government did not intervene in the pricing, and nor did it offer support. Robert, a 60-year-old male retiree, said:

“There was only one time that the government-financed 100 yuan meat benefits for one Hui household each year, but that happened only once. And now, there’s no continuing financial aid or other aid anymore.”

The sense of being ignored also applied to how the Hui people were represented in the mainstream media. In China, the television news sometimes reports on events in different communities, like, for example, with a competition to determine ‘The Best Community’. Alex, a 60-year-old male retiree, said that, as far as he could remember, there was never anyone reporting research, reports or interviews on television about Hui communities in or outside Shenyang. Therefore, he felt that the Hui people were ignored by the mainstream media, and their voices were never heard.

“There was only once that someone doing interviews about the Hui district selected a model district in the Hui community. Apart from that time, nobody else has reported anything about the Hui people or cared for the Hui people. But on television, I always see that journalists go to different communities to do interviews, but then I was thinking, why does nobody do any interviews in our Hui community? The media don’t even give the Hui people a chance to speak out our voice!”

After Alex expressed his view, the other three participants in this focus group agreed with him. Interestingly, one participant expressed a different view about why the Hui were neglected in the media. Barbara, a 49-year-old female teacher, said that this neglect was the consequence of the ‘National Unity Policy’ promoted by the Chinese government:

“Generally, there couldn’t be any health information targeting the Hui. In China, there’s one rule called ‘one national unity’. I think that you can’t distinguish different groups very clearly, as we build our school based on the core of ‘harmony’. If everything distinguished ethnic differences clearly, then this would be a big problem in society.”

In general, Barbara’s view was not shared by the other participants. Most clearly expressed a desire to be the focus of more attention from society and the media. In particular, some participants mentioned that, as the Hui population had a higher prevalence of CVRFs than other groups, there should be research conducted among them to identify the reasons for this. The participants all noted that no one had conducted research among the Hui people before.

### 3.4. The Hui’s Needs relating to CVDs

After addressing the health information they had obtained, the medical system, and general issues regarding the position of the Hui in China, the discussions focused on the needs of the participants with respect to CVD-related information. Overall, most expressed a clear need for credible, professional, and reliable information about CVDs. Lily, a 43-year-old female teacher, said:

“No matter if it’s the internet or television, when transmitting health information to people, there should be someone supervising this to ensure its credibility; we can’t accept that there’s profit behind this …We just need real and pure CVD health information.”

During the discussions, the participants were asked to list what kind of health information they needed. The analysis of their answers revealed six features. The most prominent were prevention, treatment, and general information (including symptoms, etc.) (see Table 3). Ian, a 23-year-old bachelor student, expressed the most prominent needs:

“I focus more on the information about how to prevent CVDs, knowing the mechanism of this disease; then I can adjust my eating and living habits to prevent it. If I get the disease one day, I can understand my situation based on the symptoms in the early stage before the serious symptoms come; then I can keep all this in mind and know which stage I am at myself.”

The participants thought that professional public health institutes or agencies should have programs to disseminate health information. This is because they thought that health information from such bodies was much better and more credible than that from the internet and television. The participants experienced a lot of stress due to their work and emphasized that it was impossible for them and other Hui people to devote much of their scarce leisure time to obtaining reliable health information. Olivia, a 60-year-old female laboratory technician, said:

“Like now the Hui’s prevalence of CVDs is high, so I suggest taking some action in promoting health information about CVDs among the Hui people. Do more CVD health information promotions, let Hui people get attention, and cultivate Hui people. Hui people’s education level is low, so we really need effective CVD health information.”

The analysis of the transcripts also revealed two other needs related to CVDs. The first concerned healthy and safe food, which was mentioned by two participants. They revealed that food products in China were subjected to extensive applications of fertilizers and other additives. The participants, therefore, expressed their concerns about food safety issues. Emma, a 75-year-old retired woman, said:

“The main issue is that the food people eat in China is very unsafe. Chinese people can get hypertension easily after eating food with too many additives …In China, there are too many fake things, especially the vegetables; there are too many bad ingredients added. This morning I saw the news reporting that amounts of glue were added to unborn things; I don’t think that Chinese people will feel healthy after eating food like this.”

The second need concerned financial support for insurance. Four participants, who were over 60 years old, said that they needed more financial help with their social health insurance cards. In China, people who have jobs will have these cards, which can be used to buy medicines. The government distributes a certain amount of money to the account each month, with the amount varying for different jobs and how long someone has worked. Four participants revealed that the financial help available was very different in China; for example, there were jobs like civil servants, which were famous for being ‘lifelong secure jobs’ with good benefits, and these workers perhaps had a certain amount of insurance money left each month. Meanwhile, for people with ordinary jobs, the insurance money was inadequate, according to the participants. Moreover, they had no other resources to pay for medication. Robert, a 60-year-old male retiree, said:

“The medical insurance card gives us a certain amount of money each month, but if we want to recuperate well, we can only buy some medicine this month, and then we will use next month’s money to buy the rest. If we want to follow health advice, our economic conditions don’t allow us to do so.”

Nancy, a 48-year-old female accountant, mentioned a new department in a hospital for the ‘preventive treatment of diseases’, which refers to doctors taking measures to prevent patients from becoming ill. She hoped that the government would provide financial aid to help more people in this regard; for example, if a treatment costs 100 yuan, the government can subsidize 80 yuan, meaning that people only need to pay 20 yuan themselves. If this occurred, Nancy believed that people would be more likely to make an effort to submit to treatments aimed at preventing diseases, including CVDs.

## 4. Discussion and Conclusions

This explorative research provides a preliminary understanding of health information about CVDs that the Hui people in China have successfully obtained from different sources. Building upon CCA that argues that the voices of minorities are often erased from dominant discourse [27,58,59,60], this research provides insights into what needs the Hui people have in relation to CVD health advice. It was striking that the participants mainly relied on the internet and television to obtain health information. This suggests that, although there was no information targeting the Hui in the media, the participants still consulted the general media to obtain health advice. These findings are in line with the results of previous research conducted among different ethnic groups and demonstrate that the internet is one of the main sources used when people are looking for information about a specific disease [61,62]. Minority groups that do not have access to traditional sources of health information use the internet to overcome their lack of access to specialist advice [5]. Nowadays, social media can attract individuals when it comes to acquiring health advice and play an important role in health communication [63]. In this case, WeChat has become a prominent channel for sharing and searching for health information in China [40], and was also mentioned by our Hui participants. WeChat is, thus, an important source of health information in China [40].

The Hui participants in this study experienced obstacles in getting health information about CVDs and expressed a desire for credible and professional information from reliable sources. Credibility has been described as a factor influencing message receivers’ perceptions and attitudes [64], and previous research shows that some CVD patients become anxious as a result of what they have found online [51]. On the one hand, our Hui participants had doubts about online health information and were unsure of what they could trust. On the other hand, they often distrusted the information provided by physicians, which differed from previous findings that individuals have a high level of trust in the health advice provided by doctors compared to all other sources [61]. Their distrust of physicians was not related to the ethnic difference between the Han and the Hui. Moreover, it did not generalize to the entire medical profession. It was rather the opposite because our participants generally acknowledged the importance of medical expertise and wanted to benefit from it.

Our second research question invited the focus group participants to discuss their needs with respect to health information concerning CVDs. Compared to previous studies, which suggested that information and emotional support were important to individuals with illnesses [65,66], our research found that the Hui participants talked exclusively about their cognitive need for CVD health advice. Emotions were absent in the group discussion: No one mentioned their emotional needs. Nevertheless, the participants’ need for health advice with respect to treatment for CVDs corresponded with previous findings [30,45,46,47,48,49,50]. In addition, the participants pointed out two needs that were not directly related to health information in a strict sense, namely the desire for more financial help with their social health insurance cards and the need for healthy and safe food. With respect to the wider context of health in China, the Hui participants were concerned about the lack of health information for the Hui, and they also expected mediated health-communication programs specifically targeted at the Hui.

These results indicate the value of this focus group study. We successfully reached out to the Hui minority in Shenyang City, making our study the first to use qualitative methods to explore the Hui minority’s needs regarding health information about CVDs. Meanwhile, a few limitations need to be considered when generalizing and applying findings from this study. First, our current research had a relatively small number of participants, and most of our participants’ education levels were above the average. Second, the existing relationship among participants may affect the group dynamics in the discussions. A focus group is especially effective when the participants know each other [55,56]. Some of our participants were colleagues in the same school. On the one hand, it was easy for the moderator to build rapport and facilitate the discussions; on the other hand, some participants might feel reluctant to share or intentionally withhold certain information due to the professional relationship they had with each other. Overall, the discussions in the focus groups were rich, open, and active. The focus group discussions provided meaningful and new information on the Hui minority’s unique experiences of health information seeking and health practices. Third, this research was conducted in only one city in Northeast China. The characteristics of local context and regional differences should be taken into consideration when generalizing or applying findings from this study to different contexts. Future research can compare the Hui people in Hui autonomous areas in Northwest China with their counterparts in non-autonomous areas on how they receive, process, and apply CVD-related health information. Drawing from initial findings from our qualitative research, future research could apply a quantitative approach to examine the associations between the Hui’s health information seeking behaviors and health outcomes.

In today’s culturally diverse world, intercultural communication is increasingly important [67,68]. Integrating culture in health information programs and materials for specific groups is a way to take diversity into account [13]. It will also help to enhance the effectiveness of health communication, which may contribute to eliminating the structural health inequalities present in multicultural societies [13]. The current results are also relevant for Chinese health information promoters. Indeed, they may help them to consider cultural factors and disseminate advice about CVDs more effectively to the urban Hui people. The focus group discussions showed that our participants mostly used television, the internet, and social media as their sources of information. Future health promotion campaigns should take this into account. A major difficulty in the diffusion of health information among the Hui is the distrust and feelings of neglect they expressed, as well as their fundamental need for credible, reliable, and professional health advice about CVDs. There are ethnic inequalities in health and healthcare in multicultural societies, thus healthcare systems should provide a fair service to multiethnic populations [69]. Efforts to promote health information without considering cultural dimensions are unlikely to address a specific group’s needs [70]. Researchers and health promoters alike should take the wider context of the Hui’s cultural backgrounds into consideration when developing a campaign [26]. Finally, this Chinese minority case may also be relevant for research about multicultural communication among other minority groups in other countries.

## Figures and Tables

**Table 1:** Demographics of the focus group members.

Members	Age	Gender	Occupation	Highest Education
Lisa	47	Female	Teacher	Bachelor
Wendy	53	Female	Manager	College
Lily	43	Female	Teacher	Bachelor
Jack	45	Male	Teacher	Bachelor
Nancy	48	Female	Accountant	College
Helen	39	Female	Teacher	Bachelor
Tom	43	Male	Teacher	Bachelor
Barbara	49	Female	Teacher	Bachelor
Joan	41	Female	Teacher	Bachelor
Jasmine	49	Female	Teacher	College
Emily	47	Female	Teacher	College
Mia	30	Female	Office worker	Master
Mary	35	Female	Office worker	Bachelor
Ian	23	Male	Student	Bachelor
John	24	Male	Student	Bachelor
Emma	75	Female	Retired	High school
Sara	60	Female	Custodian	High school
Olivia	60	Female	Medicine laboratory technician	High school
Robert	60	Male	Retired	High school
Alex	60	Male	Retired	High school
Ethan	23	Male	Imam	Bachelor
Eric	75	Male	Retired	High school
Thomas	57	Male	Imam	Bachelor

*Note.* All the participants’ names have been anonymized by the researchers.

**Table 2:** Sources used by the Hui participants to obtain information on CVDs.

Sources	Frequency
Internet	18
Television	14
Family and friends	10
Hospitals and doctors	10
Radio	5
Newspapers	3
Total	60

*Note.* The participants were able to write down multiple sources.

**Table 3:** The types of information on CVDs required by the Hui participants.

Types of CVD Health Information	Frequency
Prevention	17
Treatment	10
CVD general information (including symptoms etc.)	10
Healthy diets	6
Authority and government promotion	3
Medical consultation	2

*Note.* The participants were able to write down multiple types of CVD health information.
